# Characterization of *Campylobacter *phages including analysis of host range by selected *Campylobacter *Penner serotypes

**DOI:** 10.1186/1471-2180-7-90

**Published:** 2007-10-18

**Authors:** Vinni Mona Hansen, Hanne Rosenquist, Dorte Lau Baggesen, Stanley Brown, Bjarke Bak Christensen

**Affiliations:** 1 Department of Microbiology and Risk Assesment, National Food Institute, Soeborg, Denmark; 2 Department of Molecular Biology, Copenhagen University, Copenhagen, Denmark

## Abstract

**Background:**

The predominant food borne pathogen in the western world today is *Campylobacter*. *Campylobacter *specific bacteriophages (phages) have been proposed as an alternative agent for reducing the burden of *Campylobacter *in broilers. One concern in relation to phage biocontrol is the narrow host range often displayed by phages. To identify the potential of phages as a *Campylobacter *reducing agent we needed to determine their infectivity on a panel of isolates representing the *Campylobacter *strains found in broilers as well as humans.

**Results:**

In this study, *Campylobacter *phages were isolated from the intestines of broilers and ducks and from abattoir sewage. Twelve phages were investigated to determine their ability to infect the *Campylobacter *Penner serotypes commonly present in Danish poultry and patients with campylobacteriosis. A total of 89% of the *Campylobacter jejuni *strains and 14% of the *Campylobacter coli *strains could be infected by at least one of the bacteriophages. The majority of the phages infected the most common serotypes in Danish broilers (O:1,44; O:2; O:4-complex), but showed limited ability to infect 21 of the less frequent *Campylobacter *serotypes. Pulse field gel electrophoresis (PFGE) and restriction endonuclease analysis (REA) were used to characterize the phage genomes. Three categories of bacteriophages were observed. I: a genome size of ~194 kb and refractory to digestion with HhaI; II: a genome size of ~140 kb and digestible by HhaI; and III: a genome size undeterminable in PFGE. The categorization of the phages correlated with the host range patterns displayed by the phages. Six phages were subjected to transmission electron microscopy (TEM). They all belonged to the family of *Myoviridae*.

**Conclusion:**

We have characterized and identified the host range of 12 Danish *Campylobacter *phages. Due to their ability to infect the majority of the common serotypes in Denmark we suggest the phages can become an effective agent in the effort to reduce the incidence of campylobacteriosis in Denmark. This study provides the basis for future experiments in *Campylobacter *phages and knowledge for the selection of *Campylobacter *phages for biocontrol in broilers.

## Background

*Campylobacter *is a zoonotic pathogen naturally present in the gastrointestinal tract of many domestic animals and pets [1-4]. In Denmark and in many other developed countries, *Campylobacter *enteritis is the predominant food borne disease [3]. In Denmark, fresh poultry meat has been identified as the main risk factor for human campylobacteriosis [5]. Consumption of undercooked chicken meat and cross contamination with ready-to-eat foods through improper food handling contributes to human infections [6,7]. *C. jejuni *is the predominant species in broilers as well as in humans [8]. A Danish surveillance study comparing *Campylobacter *species in humans, domestic animals and food products revealed that 93% of the speciated isolates from humans were *C. jejuni*. Among these, 63% belonged to the Penner serotypes O:1,44, O:2, and O:4C (4-complex), that likewise were the most frequent serotypes isolated from broilers (46%), cattle (57%), and food (49%) [9]. *C. coli *constituted 6.5% of the human isolates. This species was occasionally found in poultry but predominantly found in pigs [9]. Strategies to improve food safety by reducing the risk of *Campylobacter *include efforts in the primary production, at the slaughter line, and at the consumer level [3]. Risk assessments have suggested that one of the most efficient ways to reduce the incidence of campylobacteriosis is through methods that reduce numbers of *Campylobacter *in poultry meat [10,11]. One such method is freezing, which has been shown to reduce the number of viable *Campylobacter *cells approximately 100 fold [12-14]. Unfortunately, frozen products do not suit the consumer demands for chilled products, that are ready to cook without thawing. Therefore, other methods to control the level of *Campylobacter *in the final product are required.

One potential strategy for controlling bacterial pathogens in food production is the application of virulent phages, viruses that can kill bacterial cells [15,16]. The phage reproduces inside the bacterial cell and cause it to lyse releasing progeny phage into the environment. Phages possess several advantages compared to traditional antibiotics, including self-replication/self-limitation and selective modification of the bacterial flora [15]. Most bacterial species are challenged by their own specific bacteriophages that seldom cross species barriers. *Campylobacter *phages have been recovered from environments harboring *Campylobacter *[17-20] and seem to be a natural part of the intestinal flora of *Campylobacter *colonized broilers [21-23]. To reduce numbers of *Campylobacter *in chicken meat, the bacteriophages may be orally administrated to the broilers before slaughter or applied onto the meat after slaughter. Studies have shown that the use of bacteriophages as biocontrolling agents significantly reduced *Campylobacter *and *Salmonella *shedding from live birds and contamination on chicken skin [24-30].

One concern in relation to commercial use of bacteriophages in the chicken production is the narrow host range displayed by these viruses [15]. The host range of *Campylobacter *phages has previously been investigated on strains representing the phage types of the United Kingdom typing scheme, phage typed reference strains of human origin, and strains isolated during fieldwork for characterization [20,21,23,28]. Even though the work has described phages by grouping them into different classes of lytic spectra and has reported qualitative information about the phages capability of killing *Campylobacter *strains, a study combining epidemiological studies on *Campylobacter *and phage host range has been missing.

The aim of this study was to isolate and characterize *Campylobacter *specific phages from the Danish poultry production (broilers and ducks) and investigate their effectiveness against the Penner serotypes that are commonly present in poultry and humans in order to be able to select effective phages for future biocontrol.

## Results

### Isolation of phages

A total of 312 samples were collected and analyzed for *Campylobacter *phages. As shown in table 1, 222 samples were isolated from broiler intestines. Of these samples, 62,6% originated from flocks that were recorded by the Danish *Campylobacter *surveillance program to be *Campylobacter *positive. For the remaining samples (ducks and abattoir samples) the flock status where not known. Table 1 shows that the type of poultry (broiler or duck) had greater influence on the phage isolation rate than sample type (intestine or abattoir). The isolation rate from broiler samples was approximately 3%. However, phages could be isolated from approximately 50% of the duck samples, making ducks the best source for *Campylobacter *phages in this study. Of the four phages isolated from the intestine of broilers with known flock status, one phage (F14) was isolated from a flock with a *Campylobacter *negative status and the remaining 3 from flocks with a positive status. For initial phage isolation, *Campylobacter *strains NCTC 12662 and 1447 were used as hosts for all 312 samples. Toward the end of the *Campylobacter *season in Denmark (late Septemper), strain NCTC 12658 was added as an additional isolation host for the last two collected samples, and phages were recovered from both of these. NCTC 12662 supported the isolation of 80% of the phages. Strain 1447 supported the isolation of one phage, F336, which was isolated from the same sample as phage F326 (Table 1). It was found that some of the phages, isolated from three independent broiler abattoir samples, were unstable during storage, therefore they were excluded from further investigation.

**Table 1:** Overview of investigated samples and isolated phages

Origin of samples	Enrichment	Number of investigated samples	Number of phage positive samples	Names of isolated phages (Isolation strain)
Broiler intestine	No	222	4	F14 (NCTC 12662)
				F198 (NCTC 12662)
				F341 (NCTC 12658)
				F346 (NCTC 12658)
Duck intestine	No	7	3	F287 (NCTC 12662)
				F325 (NCTC 12662)
				F326 (NCTC 12662)
				F336 (Lab. no.1447)
Broiler abattoir	Yes	80	5	F267 (NCTC 12662)
				F268 (NCTC 12662)
				Three isolates were unstable under lab conditions (NCTC 12662).
Duck abattoir	Yes	3	2	F207 (NCTC 12662)
				F303 (NCTC 12662)

### Molecular characterization and transmission electron microscopy

**Figure 1:**
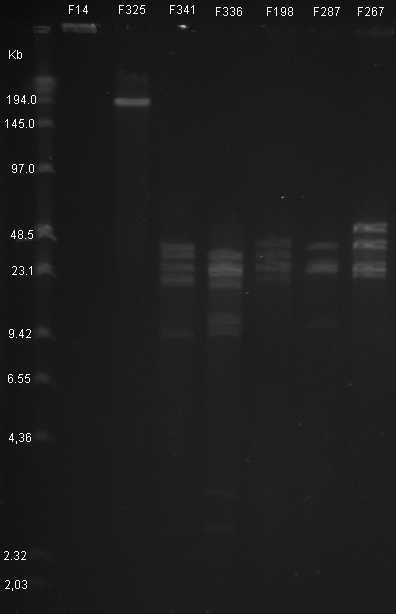
**HhaI treated genomes of phages representing the 7 REA group**. The DNA bands of phages treated with HhaI are displayed in lane 2–8. The genome of F14 does not display any DNA bands in lane 2 whereas the genome of F325 display a single band of DNA in lane 3 due to it being refractory to HhaI degestion. The next 5 lanes represent from the left to the right the HhaI restriction patterns of the 140 Kb phages (group II a-e). Concatemer (New England Biolabs, #N0350S) is seen in the first lane.

The phages were analyzed by PFGE and REA to determine genome size and sensitivity towards digestion with the restriction enzyme HhaI. Eventhough a signal were visible in the F14 well no DNA were observed to enter the the pulsed field gel, which precluded estimation of the genome size. A prolonged running time of 12 h did not change the observed outcome for phage F14. Phage F325 displayed the largest genome (~194 kb) and the remaining phages had each an estimated genome size of approximately 140 kb (Table 2). REA showed that the DNA isolated from phage F325 could not be digested with HhaI. An attempt to digest the expected F14 DNA with HhaI was unsuccesful, since DNA bands still were absent in the gel (Figure 1). All phages with a genome size of ~140 kb could be digested with HhaI. Each phage displayed between 2–5 strong bands in the PFGE ranging in size between 9–50 kb (Figure 1). The REA allowed for categorizing the 140 kb phages into five different restriction patterns (a-e) (Table 2).

**Table 2:** Phage catagories based on phage genome size and HhaI restriction patterns

Category	Genome size	HhaI Pattern (figure 1)	Phage name
I	~194 Kb	Uncut	F325~*
II a	~140 Kb	5 bands	F341~*, F346
II b	~140 Kb	5 bands + 2 weak bands	F336~*
II c	~140 Kb	4 bands	F198~*
II d	~140 Kb	3 bands	F287~, F326, F303*
II e	~140 Kb	2 bands + 1 weak band	F207, F267~, F268
III	ND*	ND	F14~*

^~^Phage used in figure 1 to illustrate the restriction pattern of the REA groups.

* Phages studied by TEM.

^#^ND, Not Determined

**Figure 2:**
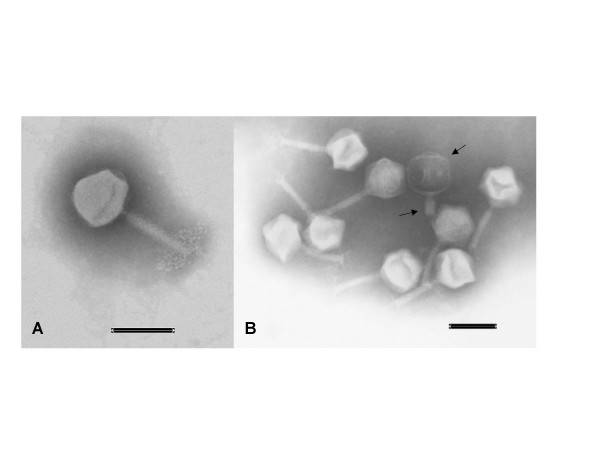
**Electron microscope images of F14 and F336**. Image A display a F14 phage. Image B display several F336 phages. The phage marked by arrows has a contracted tail and a black head, which show that its DNA has been liberated. The lower part of the tail is broken of. The Scale bars in both pictures are 0.1 μm.

Six representatives of the isolated phages were further subjected to morphological studies by transmission electron microscopy (TEM). All had icosahedral heads and contractive tails, which place them into the family of *Myoviridae *[31] (Figure 2). The morphology and sizes of the selected phages were not significantly different from each other. The length of the phages was approximately 190 nm and the average diameter of the phage heads was approximately 84 nm.

### Analysis of phage host range

The host range was investigated by the selected *Campylobacter *panel presented in Table 3. The *C. jejuni *serotypes most prevalent among human clinical isolates and broilers in Denmark were each represented by 4–5 strains, and is referred to as the common serotypes. A selection of other serotypes were represented by one strain each, and are referred to as the less common serotypes. In total the phages were tested on 24 different serotypes of which seventeen were *C. jejuni *and seven were *C. coli*. Of the *C. jejuni *strains tested in spot test 89% could be lysed by at least one of the phages, whereas only 14% of the *C. coli *strains could be lysed. The phages examined were generally well suited for infecting the *Campylobacter *strains within the serotypes O:1,44, O: 2 and O:4C, but showed less virulent towards the other *C. jejuni *serotypes tested, with exception of serotype 5j (isolation strain NCTC 12662) and serotype 23,36 (strain 81–176) that could be eliminated by 10 and 5 phages, respectively. The widest phage host range was achieved by phage F14, which affected 21 of the 34 tested *Campylobacter *strains, or 12 of the 24 serotypes. Phage F325 had a diverging host range by being most effective in eliminating the *Campylobacter *strains different from the O:1,44; O:2 and O:4C serotypes.

**Table 3:** Phage host range investigated by spot test

			Reaction of spot test*											
			
Bact. nr.	Source	Serotype and subspecies	F14	F198	F207	F267	F268	F287	F303	F325	F326	F336	F341	F346
NCTC 12658	nk^	1,44^#^	+	+	+	+	+	+	+	-	+	+	+	+
2486	Human	1,44^#^	+	+	-	+	+	+	+	-	+	+	-	-
2955	Chicken	1,44^#^	+	+	+	+	+	+	+	+	+	+	-	-
104–733	Chicken	1,44^#^	+	-	-	+	+	+	+	-	-	+	+	+
11.168	Human [36]	2^#^	+	-	-	-	-	-	-	-	-	+	+	+
1677	Chicken	2^#^	+	+	-	-	-	+	+	-	-	+	+	+
2453	Food	2^#^	+	+	-	-	+	+	+	-	+	+	+	+
2466	Chicken	2^#^	+	+	-	-	+	+	+	-	+	+	+	+
3024	Human	2^#^	-	+	-	+	+	+	+	-	+	+	+	+
1590	Human	3^#^	+	+	+	+	+	+	+	-	+	+	-	-
1447	Chicken	4c^#~^	+	+	+	+	+	-	+	-	-	+	+	+
2467	Chicken	4c^#~^	+	+	+	+	+	+	+	-	+	+	+	+
2469	Chicken	4c^#~^	+	+	-	-	-	-	-	-	-	-	+	+
2471	Chicken	4c^#~^	+	+	+	+	+	+	+	-	+	+	+	-
NCTC 12662	nk^	5j^#^	+	+	+	+	+	+	+	+	+	+	-	-
1674	Chicken	6,7^#^	-	-	-	-	-	-	-	-	-	-	-	-
2481	Chicken	11^#^	-	-	-	-	-	-	-	+	-	-	-	-
3148	Human	12^#^	-	-	-	-	-	-	-	+	-	+	-	-
2464	Chicken	18^#^	-	-	-	-	-	-	-	-	-	-	-	-
1660	Human	19^#^	+	-	-	-	-	-	-	+	-	-	-	-
2948	Human	21^#^	-	-	-	-	-	-	-	-	-	-	-	-
81–178	Human [38]	23,36^#^	+	-	+	+	+	-	-	+	-	-	-	-
1927	Human	31^#^	+	-	-	-	-	-	-	-	-	-	-	-
2462	Chicken	33^#^	+	-	-	-	-	-	-	-	-	-	-	-
2129	Human	37^#^	+	-	-	-	-	-	-	-	-	-	-	-
2144	Human	42^#^	+	-	-	-	-	-	-	+	-	-	-	-
1260	Human	53^#^	+	-	-	-	-	-	-	+	-	-	-	-
1680	Chicken	20^¤^	-	-	-	-	-	-	-	-	-	-	-	-
2476	Chicken	26^¤^	-	-	-	-	-	-	-	-	-	-	-	-
2454	Food	30^¤^	-	-	-	-	-	-	-	-	-	-	-	-
1669	Human	34^¤^	-	-	-	-	-	-	-	-	-	-	-	-
2458	Chicken	49^¤^	-	-	-	-	-	-	-	+	-	-	-	-
2474	Chicken	56^¤^	-	-	-	-	-	-	-	-	-	-	-	-
2460	Chicken	59^¤^	-	-	-	-	-	-	-	-	-	-	-	-

Total	21	13	8	11	13	12	13	9	10	15	11	10		

* +, indicate positive reaction in the spot test;-, indicate negative reaction in the spot test.

^nk, Not Known.

^# ^Belong to the *C. jejuni *sub species group

^¤^Belong to the *C. coli *sub species group.

^~ ^The serotype 4c (4-complex) includes *C. jejuni *strains that reacts with one or more of the following antisera: 4, 13, 16, 43, 50, 64, 65 in the Penner serotyping system [9].

## Discussion

Broilers have for other investigators been a fertile source for *Campylobacter *phages [21-23,28]. In this study *Campylobacter *phages were isolated much more frequently from ducks than from broilers, indicating that our method was best suited for the former purpose. Several factors can influence the harvest of phages from a particular source. The choice of host is important, since the narrow host range characteristic of most phages can cause them to remain undetected in the screening process. To overcome this obstacle, the *C. jejuni *strain NCTC 12662, known from the British phage typing system to be sensitive towards a broad range of phages [32], was chosen as a host in this study. Even though the NCTC 12662 strain, with regard to Penner serotype, is not common in the Danish broiler production, most of the isolated phages were detected by this strain [9]. However, the frequency of phage isolation from broilers was lower than previously reported from the United Kingdom [20]. The two other *Campylobacter *strains used for phage isolation (1447 (serotype O:4C) and NCTC 12658 (serotype O:1,44)) represent common serotypes in Danish poultry. However, although all samples were screened on strain 1447, only one phage was isolated. This is surprising since 1447 proved sensitive to most of the phages. Two samples were screened for phages on NCTC 12658, and phages were isolated from both, indicating that an extended use of NCTC 12658 possibly would have resulted in more isolated phages. Unfortunately, this strain was only included in the last two samples. Finally it should be emphasized that only 62,6% of the total investigated broiler flocks were *Campylobacter *positive. This is likely to have had a negative impact on the quantity of phages isolated, since presence of a phage depends on the presence of a host in the environment.

Our investigation, as well as results reported by other investigators indicate that a genome size of ~140 kb is common for *Campylobacter *phages, even though *Campylobacter *phages with genomes ranging from 110 kb to 320 kb have also been described [20,21,23,33]. Despite several attempts, phage F14 was the only phage that failed to provide DNA bands in this study, and thus the genome size could not be determined for this phage. Unfortunately, we have not been able to clarify the reasons for this result. A signal, which could be due to the presence of nucleic acid or proteins, could be observed in the F14 well after running (Figure 1). However, if this were DNA we would expect some DNA movement into the gel, irrespective of genome size of the phage DNA. We propose that the result can be due to lack of phage capside degradation during the preparation, which would result in a lack of free DNA in the well. It should be noted that *Campylobacter *phages that are unable to produce visible DNA bands have previously been described by Atterbury and co-workers [20].

Based on genome size and REA the phages were divided into three categories. I: Phages with genome size of ~194 kb and refractory to digestion with HhaI (F325); II: Phages with genome size of ~140 kb and digestible by HhaI (F198, F207, F267, F268, F287, F303, F326, F336, F341 and F346,); and III: phage genome undetectable in the PFGE (F14). Phages within each of these categories also displayed considerable differences in *Campylobacter *host range. The category I phage infected mainly the less common serotypes in Danish poultry, category II phages mainly infected the common serotypes O:1,44; O:2, O:4C, and finally the category III phage had a broader host range and infected both strains with common serotype and some of those with a less common serotype. The ~140 Kb phages digested by HhaI could be further classified into five groups having different REA patterns (a-e). There were no indications of special band patterns being connected to a particular source of isolation. All together we can distinguish between seven types of phages within the twelve isolated phages.

TEM analysis of six phages, each representing one of the types of phage found in this study, showed that the phages all belonged to the family *Myoviridae *and were of the same size as previously reported for *Campylobacter *phages [20,21,28,33].

The narrow host range often displayed by bacteriophages has been emphasized as an advantage in phage therapy, compared to chemical antibiotics, due to the limited adverse effect on the natural bacteria population [15]. On the other hand, a phage or a cocktail of phages marketed for commercial use should at least reduce the majority of the species to be controlled. The host range of category II and III phages reveals that they have potential to infect and lyse the Penner serotype O:1,44; O:2 and O:4C. A product aimed at these serotypes will potentially be capable of reducing the *Campylobacter *infections in approximately 60% of the infected broiler flocks in Denmark [8,9]. In laboratory studies it is possible to reduce *C. jejuni *infections in broilers by 1–5 logs [28,30]. This is a reduction that can cause a considerable decrease in human infections [10,11]. It will be possible to enhance the host range by producing a mixture of several phages, for example by mixing phages from each of the three categories mentioned above.

Coward et al. [34] have suggested that phage sensitivity in *Campylobacter *strains might in some cases be connected to the capsular polysaccharide (CPS) of the cell, which also is the serotypic determinant of the Penner heat-stable serotyping system [35]. In our study there seem to be some correlation between phage sensitivity and serotype, especially for group II. However this correlation is not conclusive.

## Conclusion

We have characterized and identified the host range of 12 Danish *Campylobacter *phages. Due to their ability to infect the common serotypes in Denmark we suggest the phages can become an effective agent in the effort to reduce the incidence of campylobacteriosis in Denmark. This study provides the basis for future experiments in *Campylobacter *phages, and knowledge for the selection of *Campylobacter *phages for biocontrol in broilers.

## Methods

### *Campylobacter *strains and cultures

Lawns of *C. jejuni *NCTC 12658 and NCTC 12662 from The National Collection of Type Cultures in the UK, and strain 1447, a Danish broiler isolate from our own strain collection, were used as hosts for phage isolation. A panel of 34 Penner serotyped *Campylobacter *strains were selected for host range testing. These comprised strains from a national surveillance study (11 strains from human feces, 16 strains from broiler feces, and two strains from chicken meat [8]). The remaining five *Campylobacter *strains were 11.168 [36], 81–176 [37,38], and the above mentioned strains used for phage isolation.

Unless specified, *Campylobacter *were cultured on agar at 41.5°C under microaerobic conditions (5% O2, 5% H2, 10% CO2, and 80% N2) and in broth cultures at 37°C under microaerobic conditions and stirring (250 rpm). Microaerobic environment was obtained in airtight jars by the evacuation and replacement technique (Anoxomat, Mart, Netherlands).

To prepare broth cultures, a colony was subcultured onto Colombia blood agar (C- calves blood II, Statens Serum Institut, DK) for 18 h and harvested into NZCYMCaCl_2 _broth ((21.98 g/l NZCYM-broth (Sigma)) supplemented with 1 mM CaCl2 (Sigma)). Optical density was adjusted to 0.1 at 600 nm followed by incubation for 100 minutes to allow for acclimatization of the cells.

### Sampling

In the period July – September 2004 samples were obtained from five broiler slaughterhouses and one duck slaughterhouse in Denmark. Samples of abattoir waste water were collected into clean disposable jars from accessible points along the slaughter line. From selected poultry flocks two sets of intestines were randomly collected from each flock at the slaughterhouse and packed in plastic bags. It was requested that the selected flocks were *Campylobacter *positive. The samples were transported under chilled conditions to the test laboratory.

### Treatment of samples

The content from two intestinal sets per flock were pooled and mixed 1: 5 with NZCYMCaCl_2 _broth and allowed to settle overnight at 4°C. The liquid phase was hereafter centrifuged (11,000 g, 10 min), and the supernatant syringe filtered (0.20 μm, Minisart^®^) and stored in 50 ml sterile centrifuge tubes (Sarstedt) at 4°C.

As regards to waste water samples, 50 ml sample were centrifuged (11,000 g, 10 min) and the supernatant syringe filtered by a filter resistant to clogging (0.20 μm, Corning 431218). To increase the likelihood of isolating phages from the diluted faecal material in the waste water, an enrichment procedure where implemented. 40 ml of sample were enriched in 10 ml of NZCYMx5 (109.9 g/l NZCYM broth (sigma)) and added 1 mM CaCl2 (Sigma) and 0.5 ml broth culture (strain 1447 or NCTC 12662) prepared as described above. After incubation over night (125 rpm) one drop of chloroform was added to the enrichment cultures (Merck 1.02445), centrifuged (5000 g, 15 min) and syringe filtered (0.20 μm, Corning 431218).

### Isolation of phages

Phages were detected by the plaque assay method. Double-layer plates were prepared as follows. In a 15 ml centrifuge tube 0.1 ml prepared broth culture of the host strain and 0.1 ml pre-treated sample were mixed and absorption allowed for 15 min at 37°C followed by addition of 3 ml soft agar (NZCYM broth added Agar-select 6 g/l, (Sigma)) (50°C). The soft agar were poured onto a NZCYM plate (NZCYM broth added Agar-select 12 g/l (Sigma) dried for 45 min in flow hood before use (minimum airflow, CleanLAF-o-matic, VFB 1206 BS)) and allowed to set for 15 min at room temperature on a plane surface before incubation for 18 h. If the following inspection of the double-layer indicated plaques, material from a plaque was transferred by Pasteur pipette to 1 ml SM-buffer (5.8 g NaCl (Merck), 2.0 g MgSO4*7H2O (Merck), Trizma^®^hydrochloride solution 50 ml (Sigma), 5 ml gelatin 2% w/v solution (Sigma), Milli-Q up to 1000 ml [pH 7,5]. The phages were allowed to diffuse for 1 h at room temperature or over night at 4°C before they were syringe filtered (0.22 μm, Millipore, SLGV013SL). Prepared cultures of the strains *C. jejuni *NCTC 12662 and *C. jejuni *1447 were used as host strains for all samples, whereas NCTC 12658 only were used for two samples.

### Treatment of phages

Serial dilutions of each phage were prepared in SM-buffer for purification. Then 0.1 ml of the dilution was mixed with 0.1 ml prepared culture of the *Campylobacter *strain used for isolation. Absorption was allowed for 15 min before the mixture was embedded in soft agar as described above. A single plaque per sample was isolated after 18 h of incubation. Single plaque isolation was repeated three times prior to propagation to insure purity of the phages. The double-layer method was also used for propagation of the phages to obtain plates with confluent lysis. Confluent plates were flooded with 5 ml of NZCYMCaCl_2 _broth and placed at 4°C over night. The NZCYMCaCl_2 _suspension was then transferred to a centrifuge tube and centrifuged (5000 g, 15 min) prior to syringe filtering. The propagated phages were stored in cryotubes (Nunc) at 4°C. Phage suspensions were titered on the isolation strain by plating serial phage dilutions (0.1 ml) onto double-layer plates and counting plaques after 18 h of incubation. Concentration of the phage was determined as plaque-forming units (pfu) per ml on the strain used for isolation.

### Characterization of phage genomes

For pulsed field gel electrophoresis (PFGE), phage suspensions (10^9 ^pfu/ml) were mixed 1:1 with 1.4% low melt agarose (Sigma, A-4018) and poured into molding blocks. DNA was liberated from the phage capsid by gently shaking the blocks overnight (55°C, 100 rpm) in 5 ml lysis buffer (10 mM Tris (Merck, 108382), 100 mM EDTA (Merck 1.08418) [pH 7.2], 1% sarkosyl (Sigma, L5125), 0.1 mg/ml proteinase K (BioLab P8102S)). To stop the process blocks were washed for 20 min in 5 ml washing buffer [50 mM EDTA, 20 mM Tris] supplemented with 1 mM Phenylmethylsulfonylfluride (PMSF) (Sigma). The blocks were washed another 3 times in washing buffer without PMSF (Room temperature, 100 rpm). Blocks were kept at 4°C in TE-buffer [10 mM Tris, 1 mM EDTA]. For PFGE, a 4 mm slice of a block was put in the well of a 1% agarose gel (BioRad 162–0137) and the well was sealed with 0.8% low melt agarose. PFGE marker #N0340S or #N0350S (New England Biolabs) were used as concatemer. The gel was run at 180 V, 14 h, switch time 2–10 s in a Biorad CHEF II. The gel was stained with ethidium bromide for 10 min and washed in distilled water for 30 min before a photograph was taken.

For restriction endonuclease analysis (REA) a 4 mm slice of a block was cut and placed in HhaI (Biolab R0139S) in accordance with the manufacture's instructions. Then PFGE was done as described above.

### Electron microscopy imaging

Based on genomic differences, six phages (F14, F198, F303, F325, F336, F341) were selected for transmission electron microscopy (TEM). Phages (10^8 ^pfu/ml) suspended in tris-buffer (9.0 g/l NaCl, 1.21 g/l Trizma base [pH 7.4]) were centrifuged at 10,000 g for 20 min in a microcentrifuge. The pellet was resuspended in approx. 50 μl tris-buffer and a formvar carbon-coated grid was placed for 5 min on the surface of the particle suspension before being washed in two drops of H_2_O (Milli-Q, UltraPure). For negative staining the grid was placed for 2 min on a 2% sodium silicotungstate drop and air-dried before examination in a transmission electron microscope, 60 kV (Zeiss EM 10).

### Test of host range

Bacteria lawns were prepared by pouring 3 ml NZCYM soft agar containing 0.1 ml broth culture onto NZCYM plates. After the soft agar had solidified, plates were spotted manually with 10 μl drops of phage suspensions on triplicate plates. Phage suspensions were adjusted to 10^4 ^-10^5 ^pfu per spot. NZCYMCaCl_2 _broth was used as negative control. The plates were allowed to dry for 15 min at room temperature before incubation. After 18–22 hours of incubation, the effect of the phage suspensions on the lawns were investigated. A positive response was defined as a number of ≥ 20 plaques or full lysis (clear or opaque) in the spot.

## Authors' contributions

VMH designed and carried out the microbiological and molecular work. SB conceived of the study and participated in its design. BBC and HRQ participated in its design with expert knowledge. DLB participated in the cordination and preliminary phase. VMH drafted the manuscript in collaboration with BBC and HRQ. All authores read and approved the final manuscript.
